# Assessing the Impact of Bilingualism on the Linguistic Skills of Children with Autism Spectrum Disorder (ASD) in Greece: A Scoping Review

**DOI:** 10.3390/medicina60060894

**Published:** 2024-05-28

**Authors:** Angelos Papadopoulos, Alexandra Prentza, Louiza Voniati, Dionysios Tafiadis, Nikolaos Trimmis, Panagiotis Plotas

**Affiliations:** 1 Department of Speech and Language Therapy, School of Health Rehabilitation Sciences, University of Patras, 26504 Patras, Greece; nicktrimmis@upatras.gr (N.T.); pplotas@upatras.gr (P.P.); 2 General Children’s Hospital of Patras “Karamandaneio”, 26331 Patras, Greece; 3 Department of Linguistics, School of Philology, Faculty of Philosophy, University of Ioannina, 45110 Ioannina, Greece; aprentza@uoi.gr; 4 Department of Health Sciences, Speech and Language Therapy, European University, Nicosia 2404, Cyprus; l.voniati@euc.ac.cy; 5 Department of Speech and Language Therapy, School of Health Sciences, University of Ioannina, 45110 Ioannina, Greece; tafiadis@uoi.gr; 6 Laboratory of Primary Health Care, School of Health Rehabilitation Sciences, University of Patras, 26504 Patras, Greece

**Keywords:** bilingualism, autism, ASD, language, communication, social communication, Greece, Greek context

## Abstract

(1) *Background and Objectives:* This review aims to identify the latest literature on the possible effect of bilingualism on the linguistic skills of children with autism spectrum disorder (ASD) residing in Greece. (2) *Materials and Methods:* The literature was searched in the databases of Scopus and PubMed by selecting articles and by reviewing four studies published in peer-reviewed journals. This Scoping Review is based on the standards of PRISMA recommendations for scoping reviews, while the PCC framework was used as a guide to construct clear and meaningful objectives and eligibility criteria. (3) *Results*: The publications included in the review addressed a variety of language-related skills, including morphology, the syntax–pragmatics interface, narrative ability, as well as both receptive and expressive language skills. (4) *Conclusions*: Three out of four studies provide evidence that bilingual ASD children are not disadvantaged compared to monolingual peers but rather enjoy some benefits, to a certain extent, due to bilingualism. However, the number of the reviewed studies as well as the limitations of the studies themselves render this conclusion tentative. Additionally, the findings set guidelines that speech therapists, educators, psychologists, and doctors in the Greek context need to follow when treating or educating bilingual children with ASD.

## 1. Introduction

Bilingualism has gained more attention due to the increasing number of bi-multilingual persons worldwide [1]. Bilingualism is a very complex phenomenon with many definitions been proposed through the years ranging from definitions like that of Bloomfield in 1933, p. 56 [2,3], who defines bilingualism as “native-like control of two languages,” and Haugen in 1953, who proposes that the production of “complete and meaningful utterances in other languages” is prerequisite for an individual to be considered bilingual to broader and functional definitions like those of Grosjean [4], who defines bilingualism as the use of two or more languages in everyday life. For the purposes of this review and according to the criteria set by the studies that we selected, bilingual individuals are those who have been exposed to both languages early in life (i.e., pre-school age). The human capacity for language acquisition is a remarkable ability that underlies the development of various cognitive and social skills throughout the lifespan [5]. Linguistic development is expected to substantially impact the children’s cognitive and communicative abilities, particularly in the context of engaging with peers and participating in structured educational settings [6]. Language plays a significant role in the articulation of ideas and sentiments, whereas verbal communication serves as a means to express speech [6]. Language ability is organized into five levels (phonology, morphology, semantics, syntax, and pragmatics [7]. Phonological ability, which encompasses phonetics, pertains to a language’s auditory aspects, including producing sounds, perceiving them, and understanding their function [7]. Morphological ability refers to interpreting the internal structures of words as well as the function of the morphemes, while semantic ability refers to understanding the meanings of words, phrases, and sentences. Syntactic ability is the ability to form and understand longer linguistic units, phrases, and sentences, while pragmatic ability refers to the ability to produce and understand language in the social/cultural context [8]. In addition to verbal communication, the development of social pragmatics communication comprises nonverbal and pre-verbal skills, including but not limited to eye contact, communicative gestures, turn-taking, and collaborative engagement [7].

In today’s world, bilingualism and multilingualism are the norm and not the exception. According to a systematic review study, it has been estimated that roughly half of all humans speak two or more languages [9]. Many researchers suggest that bilingualism offers several linguistic and cognitive advantages to individuals (see, for example, Bialystok, 1999, as well as the work of Van den Noort et al., 2019, for an overview) [10,11]. According to the proponents of the “bilingual advantage”, bilinguals always have both languages active [12,13]. Therefore, bilinguals must exercise greater cognitive and, more precisely, executive control over their two active language system in order to select the most appropriate language form for any given situation [9,12,13]. In recent work, the benefits of bilingualism are attributed to the efficiency in the use of attentional control [11]. Specifically, the main factor in this study is that the difference in behavior between monolingual and bilingual individuals results from differences in the effectiveness and application of attentional control between the two language groups [11]. To support this argument, the researchers present how attentional control offers a more comprehensive explanation for many observations that cannot logically be assigned to inhibition [11].

Children with autism spectrum disorder (ASD) have been shown to face challenges both in the structural as well as in the functional use of language [14] (Reindal et al., 2021). They may also encounter challenges acquiring receptive and expressive language abilities, exhibiting performance levels below the age-appropriate norms [7]. Furthermore, relevant studies [15,16,17] revealed that the development of receptive language skills is generally comparatively slower than the development of expressive language skills among children with ASD. Individuals on the autism spectrum may experience stereotypical behavior as early signs [18,19], delays in developing their lexical, syntactic, pragmatic, and semantic abilities, in addition to phonological processing delays [20]. Moreover, it should be noted that there exists considerable heterogeneity in the progression of speech development among individuals with ASD. Notably, it has been observed that speech development in this population is characterized by both delays and deviations from the developmental milestones generally observed in neurotypical children [7], especially in preschool ages (Reindal et al., 2021) [14]. Given that children on the autism spectrum face difficulties both in the structural and functional aspects of language, with the latter persisting even during the school age, they may encounter communication difficulties [21]. They also have fewer chances to fully develop their linguistic abilities because of their social communication problem. 

The DSM-5 [22] criteria for the diagnosis of ASD include (a) severe impairments in social communication and social interaction and (b) restricted and repetitive patterns of behavior, interests, or activities, as well as language skills and linguistic development, that fall within the diagnostic criteria for ASD (Criterion A-2 mentions verbal communication) [22]. Conversely, the International Classification of Diseases (ICD), 11th edition, of the World Health Organization, recognizes the significance of language use in determining clinical ASD subgroups since the presence or absence of functional language is considered decisive [23]. Therefore, ICD 11 [24] exploits language use and intellectual development [23] and categorizes ASD into five different types: (a) ASD with a disorder of intellectual development and with impaired functional language, (b) ASD with a disorder of intellectual development and with absence of functional language, (c) ASD without disorder of intellectual development and with mild or no impairment of functional language, (d) ASD with a disorder of intellectual development and with mild or no impairment of functional language, and (e) ASD without disorder of intellectual development and with impaired functional language. 

Based on officially recorded data, the proportion of bilingual children in Greece approaches 10% of the total student population. In almost every school classroom, 1–4 children speak Albanian, Russian, Turkish, or Romani, among others, as their first language or mother tongue. In 2002–2003, the estimated number of bilingual children was 96,526 (2004: 58) [25]. In 2020, due to the refugee crisis, the number of children with other first languages due to their refugee/immigrant background reached 31,000 (UNICEF, 2019). In the 2020–2021 school year, the majority of the bilingual children spoke Albanian as their home language (71%) [26]. Despite the rate of ASD children being 1.2% in 2009 [27], there is a lack of studies on the current situation of ASD bilingual school-age children [28].

A recent study in the USA by Trelles and Castro [29] reported that ¼ of the children with ASD live in a bilingual environment. Moreover, research on the effect of bilingualism on the development of language skills in children with ASD reports mixed results [9]. Parents often worry that raising a child with ASD as bilingual may increase the risk of a delay in language development. Additionally, there are no set guidelines either in Greece or internationally that speech therapists, doctors, and educators need to follow when treating or educating bilingual children with ASD. Conversely, there exist conflicting approaches and results on the matter, which place the children at a disadvantage [29]. To the best of our knowledge, there is a research gap as regards the existence of a comprehensive review study in the Greek context with Greek-speaking ASD bilinguals which could shed light on the issue of whether and how bilingualism affects the linguistic skills of ASD children. 

Specifically, our scoping review was undertaken to supplement the existing literature by addressing the following research questions: What are the effects of bilingualism in ASD Greek-speaking children as regards their language skills? Will bilingual ASD children differ from monolingual ASD children in the Greek context? Furthermore, this scoping review aims to provide researchers with the latest and most comprehensive source of information on the topic, hence facilitating its use in clinical practice. Moreover, it would be a valuable educational article since it pulls many pieces of information together and brings practitioners up to date with certain clinical aspects. In addition, this scoping review helps present a broad perspective on the impact of bilingualism in language in children with ASD in Greece.

## 2. Materials and Methods

### 2.1. Databases and Search Strategies

**Figure 1:**
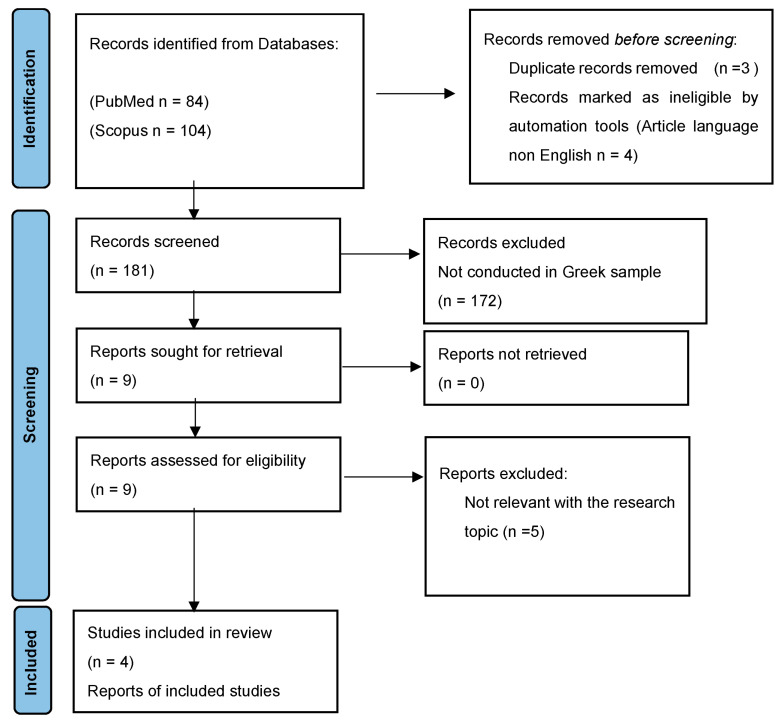
PRISMA ScR flow diagram.

The literature search was conducted in the databases of Scopus and PubMed in November 2023 by selecting articles published in relevant journals. For the search, the following keywords are used: “autism”, “ASD”, “bilingual”, “multilingual”, “children”, “communication”, “language”, “communication”, and “disorders” (see Table 1). The next step was to read all the abstracts to confirm that the studies were conducted in Greece, given that we targeted the Greek context. The dataset of the current study spanned without a time limit to include all the available literature. This Scoping Review is based on the standards of PRISMA recommendations for scoping reviews (PRISMA-ScR), and a flow diagram of the process is presented in Figure 1 [30]. The PCC framework (population, concept, and context) was used, as recommended by the literature, as a guide to constructing clear and meaningful objectives and eligibility criteria for this scoping review (Table 2) [31]. 

### 2.2. Eligibility Criteria

Specific eligibility criteria were applied to include studies in the current review. These were the following: (1) to present original data, (2) to examine bilingual children with ASD, (3) to target language and communication deficits, (4) to be written in English, (5) to provide a language assessment for children diagnosed with ASD and grow up in a bilingual family, (6) either standardized or nonstandardized measures of language ability to be used during assessment, (7) bilingualism to be defined as the use of two or more languages spoken in the home environment. 

The exclusion criteria were the following: (1) no original data (letters to the editor or other reviews were excluded), (2) the study was part of a thesis/dissertation, (3) the study targeted only ASD, and (4) case studies/reports. 

Following database screening, 84 Scopus and 104 PubMed titles and abstracts were reviewed to verify the inclusion criteria. An additional literature search was conducted for related references included in the manuscripts. After three duplicates were removed, the suitability of the scanned abstracts was assessed by two independent individuals. Then, the full texts were retrieved and read, ensuring that they met the eligibility criteria for this review. Conflicts were resolved after a broad discussion between the authors. Following this, the results of the studies were compiled and presented in tables. Mendeley reference manager software 2.114.0 was used to remove duplicates. A total of 9 articles were obtained from the search procedure, while 172 articles were excluded as the research was not conducted in a Greek sample.

### 2.3. Quality Assessment

Assessing the quality of the included papers in scoping reviews is not mandatory [32,33,34,35]. However, in accordance with the instructions provided in the Cochrane Handbook, we have included a quality assessment in our study that specifically identifies the possibility of bias in each study that we would review, and this could be considered a reflection of the overall quality of the studies [35,36]. Specifically, in Table 3, we included factors such as limitations, funding sources, and declarations of interest. 

## 3. Results

### 3.1. Summary Presentation of Studies

Of all the screened studies, four were conducted in Greece with Greek–Albanian bilinguals with ASD. Specifically, for bilingual children with ASD, three studies recruited only Greek–Albanian bilinguals [37,38,39], while only one study recruited bilinguals of other language pairs [40]. The total number of bilingual children with ASD was 97 across the studies reviewed. Regarding the gender of the bilinguals with ASD, 70 were males, while 27 were females. All the studies included a monolingual Greek-speaking control group. In the field of language assessment, the included studies employed direct and indirect measures to evaluate language and communication abilities. Furthermore, all the studies used the Children’s Expressive Vocabulary test (Renfrew, 1995) as a measure of language development adapted in the Greek language (see, for example, Vogindroukas et al., 2009, for the adaptation of the test in Greek) [41]. 

The language aspects and language-related skills assessed included morphology (e.g., derivative and inflectional morphology), spelling, pragmatics, syntax, the syntax–pragmatics interface (referential expression use, pronoun resolution skills), narrative ability (e.g., length of utterance, lexical diversity, and story length), and both receptive and expressive language skills. 

Two studies reported that bilingual children with ASD had an advantage with respect to narrative ability (e.g., micro- and macro-structure in storytelling) and socialization skills [38,40]. However, bilinguals with ASD were reported to face difficulties in punctuation, unlike bilinguals with DLD [37].

Additionally, it is important to note that in three of the studies, the data show that bilingualism did not affect children with ASD negatively as regards their language skills, despite the fact that the tests administered to the children were in the Greek language [38,39,40]. Interestingly, bilingualism was not found to disadvantage ASD children with respect to pronoun resolution skills [39], which is a complex phenomenon that lies at the interface between syntax and pragmatics. Table 4 presents information on the studies reviewed and on the obtained results.

Regarding the methodology, all the studies [37,38,39,40] used the same screening tools, such as WISC III and ADI-R, to confirm the diagnosis of ASD and were approved by the Research Ethics Committee of the Greek Ministry of Education. In addition, simultaneous bilinguals were used in the sample, since the children were exposed to both languages from birth, and the children’s bilingual experience was documented through a comprehensive parental questionnaire that was delivered from all the studies [37,38,39,40]. 

Three studies [37,39,40] tested the children in a specific order, while Andreou et al. [38] presented the tests in a randomized order. Moreover, in the four reviewed studies, the children were tested individually in a quiet area of their home, the participants completed the tasks in a single session, and the assessment material was administered in Greek, which is the social dominant language, as well as the school language [37,38,39,40]. 

Furthermore, the studies had different objectives. Skrimpa et al. [39] aimed to investigate the effects of bilingualism on pronoun resolution skills, while Peristeri et al. [40] explored narrative and nonverbal executive function abilities. Peristeri et al. [37] investigated whether spelling errors, stress assignment, and punctuation errors differ across children with developmental language disorders, ASD, and typically developing bilingual children. Finally, Andreou et al. [38] investigated the language–cognition interface in ASD by exploring whether bilingualism can enhance skills relevant to Theory of Mind, as well as whether possible positive effects would be due to executive function or syntax, and whether routes to mentalizing would differ between bilinguals and monolinguals on the spectrum.

Another difference between studies was that the experimental groups in Peristeri et al. [40] involved different mother tongues, such as speakers of Albanian, English, Russian, Swedish, German, and Ukrainian. In contrast, the other studies [37,38,39] involved only Greek–Albanian speakers. 

Three studies consisted of samples of bilingual children with autism compared to monolingual children with autism [38,39,40]. One study [37] recruited bilinguals with Developmental Language Disorder, bilinguals with ASD, and typically developing bilingual children.

As a general comment on methodological aspects of the studies, it could be supported that most were conducted with the same careful and strict methodological approach. This is attributed to the fact that most of the studies were conducted by the same research team. The studies met the ethical criteria as they were all approved by the Research Ethics Committee of the Greek Ministry of Education. 

### 3.2. Quality Assessment by Specific Factors

The most frequent limitation was the small sample size, as pointed out in two out of the four studies [37,38]. Two studies reported the lack of standardized language ability tools for the Greek language [37,38]. All studies included a control group. One study presented results that were difficult to interpret by the researchers [40]. 

Regarding funding, one study [31] reported having only open-access funding but no financial support for the research, two studies did not report information about funding, and another one reported “received no external funding.” Three of the included studies [37,38,39] declare no conflicts of interest, and one article [40] did not include information on this subject (Table 3).

## 4. Discussion

The purpose of this scoping review was to identify and analyze literature on the effect of bilingualism in ASD Greek-speaking children residing in Greece and to discuss whether bilingual ASD children fall behind monolingual ASD children as regards language skills. 

All reviewed studies employed direct and indirect measures to assess language and communication abilities. The combination of direct and indirect measures provides a more accurate depiction of children’s language skills, as direct assessment typically captures only a single moment in clinical settings [7]. In contrast, parent assessment scales can provide a longitudinal reflection in natural settings, as mentioned in the study of Gilhuber et al. [7]. 

In the study of Andreou et al. [38], the performance in the sentence repetition task was measured in terms of grammaticality and accuracy, which relates to whether the repeated sentence was grammatical and whether it was a verbatim repetition of the prompt, respectively. Both groups performed equally well in terms of grammaticality. However, the bilingual children with ASD outperformed their monolingual classmates in terms of grammaticality when producing modifying clauses, including adverbial clauses and relative clauses. As the authors mentioned, the observed pattern of results can be explained by other aspects associated with the bilingual profile and clinical characteristics of children with ASD, mainly being associated to the fact that these children had exposure to two first languages. 

In the study of Peristeri et al. [40], the correlation analyses showed that executive functions in storytelling were more actively involved in bilingual ASD children than in monolingual ASD children. As the authors discussed, global attentional biases appear to play a significant role in influencing the linguistic characteristics of narratives in ASD bilingual children, which is also observed in TD bilinguals but not in ASD monolinguals. These linguistic characteristics include lexical diversity, subordination index, use of adverbials, complexity of story structure, complement clauses, and the presence of Theory of Mind-related Inferred Speech Acts. 

The study of Skrimpa et al. [39] indicated that children with autism, whether monolingual or bilingual, have a consistent bias towards the other, extra-sentential referent across both null and overt subject pronouns. In contrast, TD monolingual and bilingual children tended to favor subject and object referents in their preferences for referring to objects. Bilingualism appeared to have a regulating impact on how autistic children interpreted null pronouns. Specifically, as the researchers explained, the ASD bilingual children showed an equal preference for the subject and other referents in discourse contexts with ambiguous null pronouns. This was in contrast to their monolingual autistic peers, who tended to identify extrasentential entities as the preferred referents.

The study of Peristeri et al. [37] indicated that the deficits in the writing abilities of individuals with DLD and ASD may have different underlying causes. Specifically, the writing performance of the ASD group is more influenced by difficulties in organizing and connecting larger units of text, whereas DLD children exhibit deficits in lower-level aspects of writing, such as spelling. As the authors discussed, the consequences of this research can be examined from two perspectives: Theoretical implications of the findings include the potential to generate theories regarding the mechanisms that contribute to writing abilities in ASD and DLD bilingual children. Additionally, the findings may help explain the distinct patterns of errors related to spelling, stress, and punctuation in these two populations.

This review analyzed studies that employed various assessment tools; for example, four studies used the Children’s Expressive Vocabulary Test in Greek adapted by Renfrew [41]. Many relevant publications [46,47] have supported that inadequate assessment tools would underrepresent bilingual children’s language abilities. Meir and Novogrodsky (2020) also discussed that researchers might find a bilingual advantage if they tested bilingual children in their socially and, crucially, individually dominant language [47]. This aligns with the criticism of the inaccuracy of single-language measures for bilinguals [48]. In this review, two studies [38,40] indicated that bilingualism allottees exhibited the language deficits attested in ASD, where bilinguals performed better than monolingual ASD children. Regarding narrative skills, the results of two studies [38,40] revealed a bilingual advantage in narrative abilities. In this light in our review, we found that bilingualism might not seem to have an adverse effect on the language skills of Greek-speaking ASD children, with these findings being in line with those of previous studies with more global scope [7,9,49]. 

As regards three previous review studies that include international literature [50,51,52], it must be noted that they discuss findings of older research. In 2015, Drysdale and colleagues conducted an in-depth review of eight studies that specifically examined bilingual language development in a sample of 182 bilingual ASD children [50]. This review [50] showed that bilingual ASD children do not display any additional delays in language development compared to monolinguals with ASD. Additionally, this review reported that bilingual children with ASD show development in both their languages, which leads researchers to support that bilingualism does not disadvantage ASD children. It is important to note that none of the reviewed studies reported any negative impact of bilingualism on the language development for ASD children. 

In 2016, a narrative study [51] aimed to review research on the effects of the age of onset of exposure to the two languages and the amount of bilingual exposure, as well as on outcomes of either direct language intervention or educational placements in three groups of children with Developmental Disabilities: Specific Language Impairment (SLI), ASD, and Down syndrome (DS). Specifically for the ASD group, three studies compared the language abilities of simultaneous ASD bilinguals to monolinguals with respect to the majority language or the language used most often in the participant’s home. All three studies found that the simultaneous ASD bilinguals performed equivalently to age-matched monolinguals with ASD on direct receptive vocabulary tests and general expressive and receptive language measures. Also, the number of English words produced by the Chinese/English simultaneous ASD bilinguals was lower than that of English-speaking monolinguals with ASD, but the bilinguals’ total vocabulary (the number of words produced in both languages) was significantly higher. These findings highlight the need to test both bilinguals’ languages to measure their vocabulary and language skills accurately. 

A systematic review was conducted by Emily M. Lund, Theresa L. Kohlmeier, and Lillian K. Durán in 2017 [52]. Seven articles met the inclusion criteria and were reviewed (all were published within the previous five years), covering various languages and involving predominantly young, simultaneous ASD bilingual children. This review addressed the following questions: (a) What are the characteristics and the quality of published, peer-reviewed research on the language development of bilingual ASD children compared to other populations? (b) How do the expressive and receptive language skills of bilingual ASD children compare to those of monolingual ASD children? A recent systematic review study in 2017 [52] reported minimal differences between young ASD bilingual children and monolingual ASD children. Bilingual children are at an advantage to a certain degree in vocabulary, while monolingual children seem to utter the first words earlier, which is expected given that bilingual children have to process two languages and not one. Although these advantages vary, monolingual children may have some potential advantages regarding receptive language, which is expected given that monolinguals have massive input and are processing only one language. Additionally, the results of the review study suggested that bilingualism does not cause significant delays in language development in bilingual ASD compared to monolingual ASD children [52]. 

Moreover, regarding the Greek and Albanian languages and their relationship, the findings showed that Greek monolingual and Greek–Albanian ASD children had similar performance in narrative skills. Since both Greek and Albanian are morphologically rich languages [53], this could have boosted children’s language processing skills in the language of the testing, that is, Greek [38]. As a result, these bilinguals managed to achieve a performance that is comparable to that of monolingual peers. The two languages share morphosyntactic properties (i.e., richness in nominal and verbal forms) as well as similar structures as regards subordinate clauses, which refers to the syntax domain. Specifically, Albanian, like Greek, is a morphologically rich language with flexible word order. Nouns are inflected for number and case, while verbs for person and number. Additionally, all the structures used in the Greek SRT exist in Albanian too (albeit may be formed differently in some cases) [54]. This could be an explanation of bilingual ASD children’s higher performance in sentence repetition tasks compared to monolingual peers.

Furthermore, one important consideration that should be acknowledged as a limitation of the studies included in our review is the extent to which the participants are accurately diagnosed as children with ASD or as bilingual children with ASD. Additionally, it is necessary to assess whether the language outcomes observed in these studies are representative of those observed in the larger population of children with ASD. The above is particularly relevant as the broader population may not have the same level of access to early diagnosis and intervention [52]. Another limitation is the small sample size of all four studies, as occurred by the quality assessment of the studies in Table 3. Finally, we could not analyze the potential impacts of sex, type of bilingualism, and method of evaluation because the examined studies were inconsistent regarding these variables. Additionally, some studies used unstandardized assessment tools to complete the assessment. 

Finally, in our review study, in three out of four studies, there is evidence to some extent that bilingualism does not disadvantage Greek-speaking ASD children with regard to their language skills, although the tests were only administered in Greek, which may not be their individually dominant or preferred language. However, due to the limitations of the studies, as pointed out by the researchers themselves and to their limited number (n = 4), these results cannot be generalized to the larger population of ASD children. However, findings like these may start challenging standard views that bilingualism disadvantages children with ASD. In addition, a current systematic review study [7], including studies worldwide, reached the conclusions similar to our aforementioned proposal. The conclusions from the previous narrative reviews lend support to our current findings and enhance our review to emerge the impact of bilingualism on deficits in children with ASD over time.

## 5. Conclusions

In conclusion, while in the past speech–language pathologists, teachers, and other service providers frequently would advise parents of children with ASD to avoid dual-language environments [4,46], the validity of these suggestions has been questioned during the last few years. Based on our comprehensive analysis, although the evidence is not very strong or conclusive, our review highlights two key findings that are relevant to our query. Firstly, ASD bilingual children might exhibit advantages compared to monolingual children with ASD in specific language tasks, but the evidence so far is not sufficient to be generalizable in the Greek sample let alone in the international sample. Secondly, studies have consistently shown a consensus that bilingualism at least does not hinder language skills in children with ASD in the Greek sample. Finally, it is crucial to note that more information on environmental factors affecting bilingual language exposure, and thus proficiency, should be included in future studies as some of the current research on Greek-speaking ASD children may be weak on this point. Additionally, it would be worthwhile to undertake a study on the cognitive abilities of bilingual ASD children and its possible effect on their language skills in the Greek context. Finally, it is necessary for researchers to conduct studies with larger sample sizes and longitudinal perspective in order to determine the long-term impact of bilingualism in the Greek context and internationally. 

## Figures and Tables

**Table 1:** Databases and search strategies used.

Databases		Results
PubMed	(((((ASD) OR (Autism)) AND (bilingualism)) AND (Children)) AND (language)) AND (communication)	84
Scopus	(TITLE-ABS-KEY (autism) OR TITLE-ABS-KEY (asd) AND TITLE-ABS-KEY (bilingualism) AND TITLE-ABS-KEY (language OR communication) AND TI-TLE-ABS-KEY (children))	104

**Table 2:** The PCC framework.

PCC Element	
Population	Children with an ASD diagnosis
Concept	What are the effects of bilingualism in ASD Greek-speaking children as regards their language skills? Will bilingual ASD children differ from monolingual ASD children in the Greek context?
Context	In Greece

**Table 3:** Quality assessment in accordance with the instructions provided in the Cochrane Handbook.

Studies	Limitations	Funding	Declarations of Interest
[37]	1. Limited number of standardized language ability tests in Greek. 2. Small sample size of the children. 3. More measures, including Theory of Mind and executive functions, could have shed more light on evaluating ASD and DLD bilingual children’s performance in written production.	Open-access funding is provided by HEAL-Link Greece. The authors received no financial support for the research.	The authors have no conflicts of interest to declare.
[38]	1. Limited number of participants. 2. Did not include data from TD children.	This research received no external funding.	The authors declare no conflicts of interest.
[39]	1. Single-language administration of the pronoun comprehension task may have masked subtle processing differences between the experimental groups, which could have affected children’s referential preferences. 2. Lack of standardized language tools in the children’s heritage language (Albanian). 3. The groups’ performance might be influenced by nonlinguistic factors, such as executive functions, inhibition, and working memory. 4. Limited sample size of the children (Not reported).	No relevant information reported.	All authors certify that they have no affiliations with or involvement in any organization or entity with any financial interest or nonfinancial interest in the subject matter or materials discussed in this manuscript.
[40]	1. Limited sample size of the children (Not reported).	No relevant information was reported.	No relevant information reported.

**Table 4:** Data Extraction and Synthesis (sample’s characteristics and basic findings of the articles included in the review).

Study	N of BwASD	Gender (M/F)	Age [Range] (Years)	Language and Communication (Social) Assessment	Language Dimensions and Communication	Country/Languages (L1–L2)	Findings
[37]	28	24/4	10; 3 [9; 1–11; 9]	1. Expressive Vocabulary test in Greek [41] 2. Writing task on A4 paper (pen and pencil testing)	Phonological, grammatical, and orthographic (spelling and punctuation).	Greece (Greek–Albanian)	Bilinguals with ASD were more challenged in punctuation compared to bilinguals with DLD.
[38]	29	20/9	10, 4 [7, 2 to 15, 6]	1. Expressive Vocabulary test in Greek [41] 2. Sentence Repetition Task (COST Action IS0804)—syntactic complexity [42] 3. Sentence repetition subtest of the CELF-4 [43]	Pragmatics, Syntax	Greece (Greek–Albanian)	In the sentence repetition task, bilinguals scored higher than monolinguals in complex sentences, specifically in adverbial and relative clauses.
[39]	20	16/4	10; 2 [8; 1 to 12; 2]	1. Expressive Vocabulary in Greek [41] 2. Online listening sentence–picture verification task [44] Sentence repetition task (COST Action IS0804) [42]	Morphology–pronoun resolution skills	Greece (Greek–Albanian)	Bilingualism was not detrimental to the autistic children’s pronoun resolution skills, suggesting that having acquired more than one language does not negatively affect ASD children’s deficits in the comprehension of pronouns.
[40]	20	20/0	[7; 7–11; 9]	1. Expressive Vocabulary in Greek [41] 2. Sentence repetition task (COST Action IS0804) [42] 3. Narrative production task [45]	Language screening for narrative ability	Greece (ten = Albanian-, three = English-, two = Russian-, two = Swedish-, two = German-, and one = Ukrainian-speaking)	1. Bilingualism enhances micro- and macro-structure storytelling skills in ASD children. 2. Bilingualism mitigates children’s detail-oriented processing style in ASD. 3. ASD Bilinguals employ a broader range of executive functions in storytelling than monolinguals.

BwASD: Bilinguals with Autism Spectrum Disorder; CELF-4: Clinical Evaluation of Language Fundamentals; PPVT-III: Peabody Picture Vocabulary Tests-III; EVT: Expressive Vocabulary Test; TLD: typical Language Developing; HFA: High-functioning Autism.

## Data Availability

Not applicable.
